# Different Wood Anatomical and Growth Responses in European Beech (*Fagus sylvatica* L.) at Three Forest Sites in Slovenia

**DOI:** 10.3389/fpls.2021.669229

**Published:** 2021-07-26

**Authors:** Domen Arnič, Jožica Gričar, Jernej Jevšenak, Gregor Božič, Georg von Arx, Peter Prislan

**Affiliations:** ^1^Department for Forest Technique and Economics, Slovenian Forestry Institute, Ljubljana, Slovenia; ^2^Biotechnical Faculty, University of Ljubljana, Ljubljana, Slovenia; ^3^Department of Forest Yield and Silviculture, Slovenian Forestry Institute, Ljubljana, Slovenia; ^4^Department of Forest Physiology and Genetics, Slovenian Forestry Institute, Ljubljana, Slovenia; ^5^Swiss Federal Research Institute for Forest, Snow and Landscape Research (WSL), Birmensdorf, Switzerland

**Keywords:** *Fagus sylvatica*, wood anatomy, tracheograms, dendrochronology, intra specific plasticity

## Abstract

European beech (*Fagus sylvatica* L.) adapts to local growing conditions to enhance its performance. In response to variations in climatic conditions, beech trees adjust leaf phenology, cambial phenology, and wood formation patterns, which result in different tree-ring widths (TRWs) and wood anatomy. Chronologies of tree ring width and vessel features [i.e., mean vessel area (MVA), vessel density (VD), and relative conductive area (RCTA)] were produced for the 1960–2016 period for three sites that differ in climatic regimes and spring leaf phenology (two early- and one late-flushing populations). These data were used to investigate long-term relationships between climatic conditions and anatomical features of four quarters of tree-rings at annual and intra-annual scales. In addition, we investigated how TRW and vessel features adjust in response to extreme weather events (i.e., summer drought). We found significant differences in TRW, VD, and RCTA among the selected sites. Precipitation and maximum temperature before and during the growing season were the most important climatic factors affecting TRW and vessel characteristics. We confirmed differences in climate-growth relationships between the selected sites, late flushing beech population at Idrija showing the least pronounced response to climate. MVA was the only vessel trait that showed no relationship with TRW or other vessel features. The relationship between MVA and climatic factors evaluated at intra-annual scale indicated that vessel area in the first quarter of tree-ring were mainly influenced by climatic conditions in the previous growing season, while vessel area in the second to fourth quarters of tree ring width was mainly influenced by maximum temperature and precipitation in the current growing season. When comparing wet and dry years, beech from all sites showed a similar response, with reduced TRW and changes in intra-annual variation in vessel area. Our findings suggest that changes in temperature and precipitation regimes as predicted by most climate change scenarios will affect tree-ring increments and wood structure in beech, yet the response between sites or populations may differ.

## Introduction

European beech (*Fagus sylvatica* L.) is one of the dominant tree species of mixed and deciduous temperate European forests (del Río et al., 2017), with a wide distribution range and thus high intra-specific plasticity (Dieler and Pretzsch, 2013). It grows on forest sites with diverse growing conditions; from moist and cold sites in the southern part of Scandinavia to dry and hot areas in the northern part of the Iberian and Apennine peninsulas (Bolte et al., 2007). Due to the wide distribution range, beech populations adjust their growth traits, such as leaf phenology and growth and frost resistance, to their specific environmental conditions (Vitasse et al., 2011). Phenotypic plasticity of beech has been extensively studied in past decades to understand the survival and future distribution range of beech under climate change (Matyas et al., 2009). Across Europe, common garden experiments were established to understand the effect of beech provenances under different environmental conditions (Robson et al., 2018). Numerous studies performed in these experiments focused on leaf phenology. A latitudinal trend in leaf flushing was observed, with northern and southern provenances being late and early flushing, respectively, although some exceptions exist (Stojnić et al., 2015). In addition, productivity and wood structure (Eilmann et al., 2014; Jezik et al., 2016; Klisz et al., 2019) or growth strategy and survival (Vitasse et al., 2010) were investigated. Although such experiments provided important insights into differences between provenances, they were usually performed on relatively young trees, which exhibit different climate sensitivity compared to older trees (Trouillier et al., 2018). Thus, studies investigating the response to changing climate in adult beech populations differing in leaf phenology in terms of xylem wood structure are rare.

Although leaf unfolding in spring coincides with the onset of wood formation in beech (Čufar et al., 2008), no clear relationship between variation in leaf phenology and radial growth was observed (Čufar et al., 2015). The variability of climate conditions prior to and during the growing season significantly affects tree growth and thus the productivity of beech forests (Di Filippo et al., 2007). Different climatic factors influence growth throughout the European beech distribution range. In southern and western Europe, high temperatures in early spring positively affected the onset of beech xylem production, which is reflected in wider tree rings while high summer temperatures showed a negative effect on growth (Di Filippo et al., 2007; Čufar et al., 2008; Lenz et al., 2013; Martinez del Castillo et al., 2018). In northern and north-eastern parts of Europe, summer precipitation and frequent spring frosts positively and negatively influenced beech radial growth, respectively (Weigel et al., 2018; Harvey et al., 2019; Muffler et al., 2020). At the limit of the eastern distribution range, annual increments in beech were positively affected by late spring precipitation and negatively by summer temperatures (Šimůnek et al., 2019). In central and south-central Europe, tree ring width in beech was positively correlated with summer precipitation while more frequent drought-induced extreme climatic events were the main negative influencing factor on growth (Stojanović et al., 2018; Garamszegi et al., 2020). The response of trees to variations in climatic conditions also differed with elevation; in contrast to low elevation sites, higher temperatures during the growing season at high elevation sites had the opposite effect on beech growth (Di Filippo et al., 2007). Moreover, future predictions based on climate change scenarios suggest that at optimal forest sites with sufficient water availability, the duration of the growing season and, consequently, xylem increments will increase in the coming decades (Prislan et al., 2019).

While several studies have been performed on the impact of the environment on beech radial growth, little is known about how intra-annual variability of temperature and precipitation affects xylem structure. However, structural adjustments of wood to environmental cues play a decisive role in defining the hydraulic and mechanical properties of wood and, consequently, tree performance, and survival (Chave et al., 2009; Fonti et al., 2010). Stojnic et al. (2013) found similar productivity rates and wood structure in provenances from mesic sites and local provenances adapted to dry conditions at the provenance trial, suggesting that provenances form mesic conditions are able to adapt to drier conditions. Eilmann et al. (2014) confirmed that beech from southern provenances respond differently to drought conditions compared to those from northern provenances, suggesting genetic control of xylem performance in beech. Little is known about the impact of drought conditions on the anatomical features of wood. Giagli et al. (2016) found higher vessel density (VD) and water conductive area in a year characterized by drought conditions. Schume et al. (2004) observed that drought conditions were responsible for reduced vessel area in poplar. Furthermore, Hajek et al. (2016) observed that beech responds to drought by adjusting vessel number rather than vessel area. Similarly, Prislan et al. (2018) recorded differences in VD between two sites with sufficient water availability but at different altitude. Unlike short-term wood formation studies, quantifying wood cell anatomical features in tree rings provides a long-term approach to monitoring changes in growth phenology, climate sensitivity, xylem functioning, and xylem plasticity (Abrantes et al., 2012; Reyer et al., 2015; Castagneri et al., 2017). The availability of this information may be critical for evaluating the range of plasticity in tree species under different environmental conditions and, ultimately, for predicting their responses to future climate scenarios.

The aim of this study was to analyze the relationships between climatic conditions, tree ring widths, and xylem vessel features in beech in three forest stands with different climatic regimes and different leaf phenology of beech (two early and one late flushing site; Kramer et al., 2017; Robson et al., 2018) for a period between 1960 and 2016. By subdividing annual xylem increments into quarters, we evaluated intra-annual variations in the vessel characteristics to understand how beech from the selected sites adjust xylem anatomy to short-term changes in growth conditions, as previously shown by Sass and Eckstein (1995), Abrantes et al. (2012), and Castagneri et al. (2017). In addition, we analyzed the influence of extreme weather events (i.e., exceptionally hot or wet summers) on vessel features within the tree rings. We hypothesized that (1) there is no relationship between tree-ring widths (TRWs) and vessel area because they contain different climatic information; (2) beech trees at the selected sites respond differently to local climatic conditions with respect to TRWs and vessel features suggesting adaptation to local conditions; (3) intra-annual vessel-climate relationship is not temporarily stable resulting in different trend of climatic signal in vessel area; and (4) at the site with the late flushing population, trees are more susceptible to weather variation than those characterized as early flushing, due to the shorter growing season.

## Materials and Methods

### Study Species, Site Characteristics, and Climate Data

The selected forest sites represent origin forest stands for three European beech provenances in Slovenia, i.e., Idrija, Javornik, and Mašun. Seed collected at these sites was previously used in European beech provenance trials, in which differences in leaf phenology, growth trends, and survival strategies were assessed (von Wühlisch, 2004; Gömöry and Paule, 2011; Robson et al., 2011); beech at the Idrija site (IDR) is a late flushing, while beech from Mašun (MAS) and Javornik (JAV) are characterized as early flushing. All studied sites are located at a similar altitude between 904 and 958 m a.s.l. and belong to the *Abieti-Fagetum* forest association (Table 1).

**Table 1:** Characteristics of the sites JAV, MAS, and IDR; representing the local site conditions, as well as sampled tree characteristics, number of trees (N), mean diameter at breast height (DBH), and mean tree height (H).

Site characteristics										Tree characteristics		
Site	Latitude	Longitude	Bedrock	Aspect	Inclination (°)	Temperature (°C)			Precipitation (mm)	*N*	DBH (cm)	H (m)
						Average	Maximal	Minimal				
JAV	45° 43' 59,09"	14° 20' 59,38"	Limestone	SW	10	7.9	13.0	2.8	1,514	17	43.0	30.0
MAS	45° 37' 59,87"	14° 23' 00,43"	Limestone	S	11	6.4	11.1	1.7	1,609	17	39.1	28.0
IDR	46° 00' 09,71"	13° 53' 52,94"	Limestone, Flint, and Sandstone	SE	10	8.4	13.2	4.2	2,090	17	43.7	27.5

**Figure 1:**
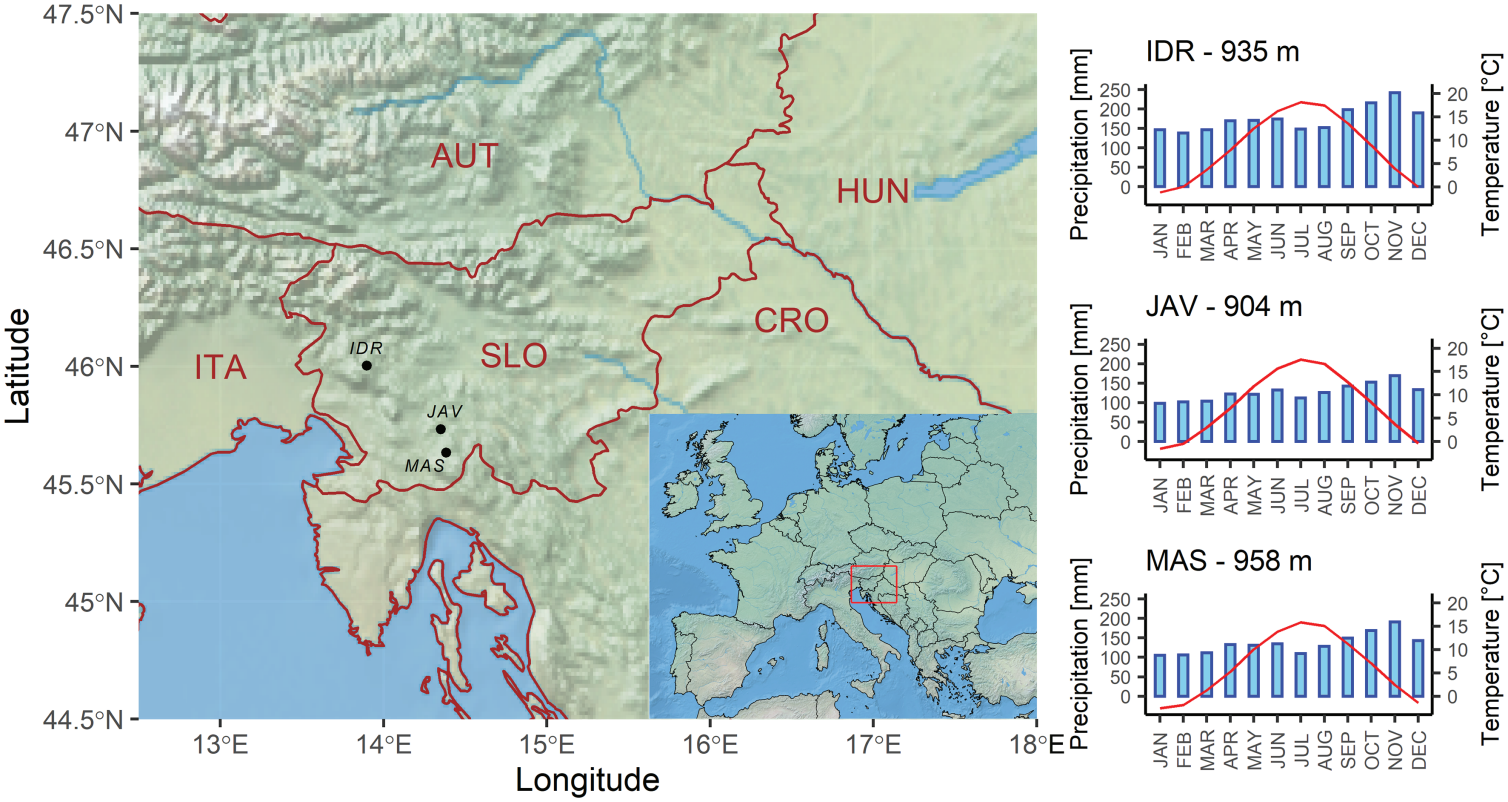
Geographical locations and climate diagrams of selected sites, i.e., Idrija (IDR), Javornik (JAV), and Mašun (MAS). Climate diagrams are based on E-OBS daily climate datasets for the period from 1960 to 2016.

We used the E-OBS daily climate data version 21e o 0.1 regular grid (Cornes et al., 2018) and extracted mean, minimum and maximum daily temperatures, and sum daily precipitation from the nearest grid point. All sites receive relatively large amounts of precipitation, i.e., between 1,500 and 2,100 mm annually, which are nearly equally distributed over the entire year, with a peak in October and November (Figure 1; Supplementary Figure S4). The E-OBS climate database refers to records from weather stations where daily climate data were collected from ground-based observation stations, operated by the National Meteorological Services between January 1950 and December 2019 (Cornes et al., 2018). Since the national weather stations located around the selected sites started recording climate data around 1960, we identified the 1960–2016 periods as optimal and used this for further analyses.

### Sampling and Tree-Ring Width Measurements

Sampling was performed at the end of August in 2016, when the xylem ring was already fully formed (Prislan et al., 2013). At each site, 17 dominant beech trees were selected, with an average diameter at breast height (DBH) of 42 ± 6 cm (Table 1). Two cores were collected per tree using a 5-mm increment borer (Haglöf Sweden, Långsele). In the laboratory, the collected cores were air-dried and fixed on wooden holders. They were then sanded to obtain a clear surface for recognition of tree ring borders. ATRICS (Levanic, 2009) was used to capture high-resolution digital images, while TRW measurements were performed using CooRecorder & CDendro software (Cybis, Saltsjöbaden, Sweden). The final cross-dating was done using PAST-5 (SCIEM, Brunn, Austria) software.

### Analysis of Xylem Vessel Features

Quantitative wood anatomy analysis was performed on six randomly selected cores per site. The random selection was used to ensure representative within-population variability rather than maximizing the climate signal. Since the same cores were used as for TRW measurements, they were first soaked in water before removal from the wooden holders. The samples were then prepared for observations under a light microscope according to the protocol suggested by von Arx et al. (2016); i.e., each core was split into segments of similar length (3–4 cm). A 15–20 μm thick transverse section was cut from each segment with a WSL sledge microtome using OLFA-80×9 mm spare blades (Gärtner et al., 2015). The sections were then treated with bleaching solution (5–15% chlorine content) to remove sawdust from the cell lumina and to improve the staining intensity in the subsequent staining with a water mixture of safranin and Astra-blue. Permanent slides were prepared using Euparal mounting medium. High-resolution images (0.514 pixel/1 μm) of the sections were prepared using a Leica DM 4000 B light microscope (Leica Microsystems, Wetzlar, Germany) at 50× magnification, a Leica DFC 280 digital camera (Leica Microsystems, Wetzlar, Germany), and LAS image analysis software (Leica Application Suite). Image-sequences of the xylem rings were captured with at least 25% of the overlapping area and then merged using PTGui v11.16 Pro (New House Internet Services B.V., Rotterdam, Netherlands). Panoramic pictures were then processed with image analysis software Image-Pro Plus 7.1 and ROXAS (v3.0.437) to obtain vessel features (von Arx and Carrer, 2014). Vessel lumen areas were measured within each ring for the period 1940–2016. Chronologies of the following parameters were then established; (1) mean vessel area (MVA), (2) VD as the number of vessels per squared mm, and (3) relative conductive area (RCTA) representing the percentage of cumulative vessel area within the measured area (the radial width of the measured area was represented by TRW, while the tangential width of the measured area was around 2 mm). Furthermore, to assess the intra-annual variability of vessel features, each vessel was assigned to one of four radial tree-ring sectors of equal width (henceforth “quarters”) based on its center coordinate and then MVA was calculated for each quarter (Q1–Q4 MVA).

### Statistical Analyses

All raw TRW series, as well as annual time series of vessel features, i.e., MVA, VD, RCTA, and Q1–Q4 MVA, were standardized using the *detrend()* function from dplR (Bunn, 2008). A cubic smoothing spline with a 50% frequency cut-off at 32 years was fitted to each individual raw series. To obtain a detrended index, the ratio between the observed and fitted values was calculated. Finally, for each parameter and site, standardized series were pre-whitened and averaged to residual chronologies by computing the robust bi-weight mean. Residual chronologies were therefore used to calculate climate-growth correlations, while raw series were compared to assess differences among sites. Please note, since we use raw and detrended chronologies, we refer to raw chronologies as TRW, MVA, VD, RCTA and Q1-Q4 MVA, while detrended chronologies are referred to by the postposition “i,” i.e., TRWi, MVAi, VDi, RCTAi, and Q1–Q4 MVAi. The quality of site chronologies was described with common descriptive statistics, such as expressed population signal (EPS), mean inter-series correlation (rbar), and Gleichlaufigkeit (%GLK; Cook and Kairiukstis, 1990).

To test the hypothesis of equal means of tree-ring characteristics, i.e., TRW, MVA, VD, RCTA, and Q1–Q4 MVA among the sites, we used parametric and non-parametric statistical tests. Repeated measures (rm) ANOVA was used when assumptions for parametric tests were met, while in other cases, the nonparametric Friedman test was applied. Similarly, subsequent pairwise comparisons for rmANOVA were assessed with Tukey’s test, while for the Friedman test; pairwise comparisons were done with the Wilcoxon test. Finally, linear regression models were used to assess the relationships between TRW and vessel features (MVA, VD, and RCTA).

Climate-growth correlations were analyzed using the *daily_response()* function from the dendroTools R package (Jevšenak and Levanič, 2018; Jevšenak, 2020), whereby day-wise aggregated correlations were calculated using 1,000 bootstrap samples considering all windows between 7 and 60 days from the previous June to the current October. Pearson correlation coefficients were calculated for the 1960–2016 period and only those with *p* < 0.05 were used to infer the relationship of climate and TRWi and vessel features (i.e., MVAi, VDi, and RCTAi within the tree rings, and in the case of MVAi also within the tree-ring quarters).

To assess the plasticity of tree-ring structure resulting from extreme weather conditions, we employed an additional comparison of standardized tracheograms (Schume et al., 2004) and compared these within three extreme dry and wet summers. Extreme years at each site were selected according to maximal or minimal amount of precipitation between the first of June and first of August, respectively. To ensure the selection of extreme years, the standardized precipitation evapotranspiration index (SPEI) was calculated (Vicente-Serrano et al., 2010). An ANOVA test was performed to test the differences of relationship between tree ring width characteristics (TRW, VD, and RCTA) and climatic conditions (maximum and minimum temperatures, precipitation amount, and SPEI) between dry and wet years. Furthermore, to develop the tracheograms, we divided the area of each vessel by the raw MVA in the particular year to account for the age-related trend (Carrer et al., 2015). We then smoothed each trend using the general additive model (GAM), which were then used in the Kolmogorov–Smirnov test to infer statistically significant differences in the intra-annual MVA distribution in years with extreme wet and dry conditions.

## Results

### Tree-Ring Width and Vessel Features

**Figure 2:**
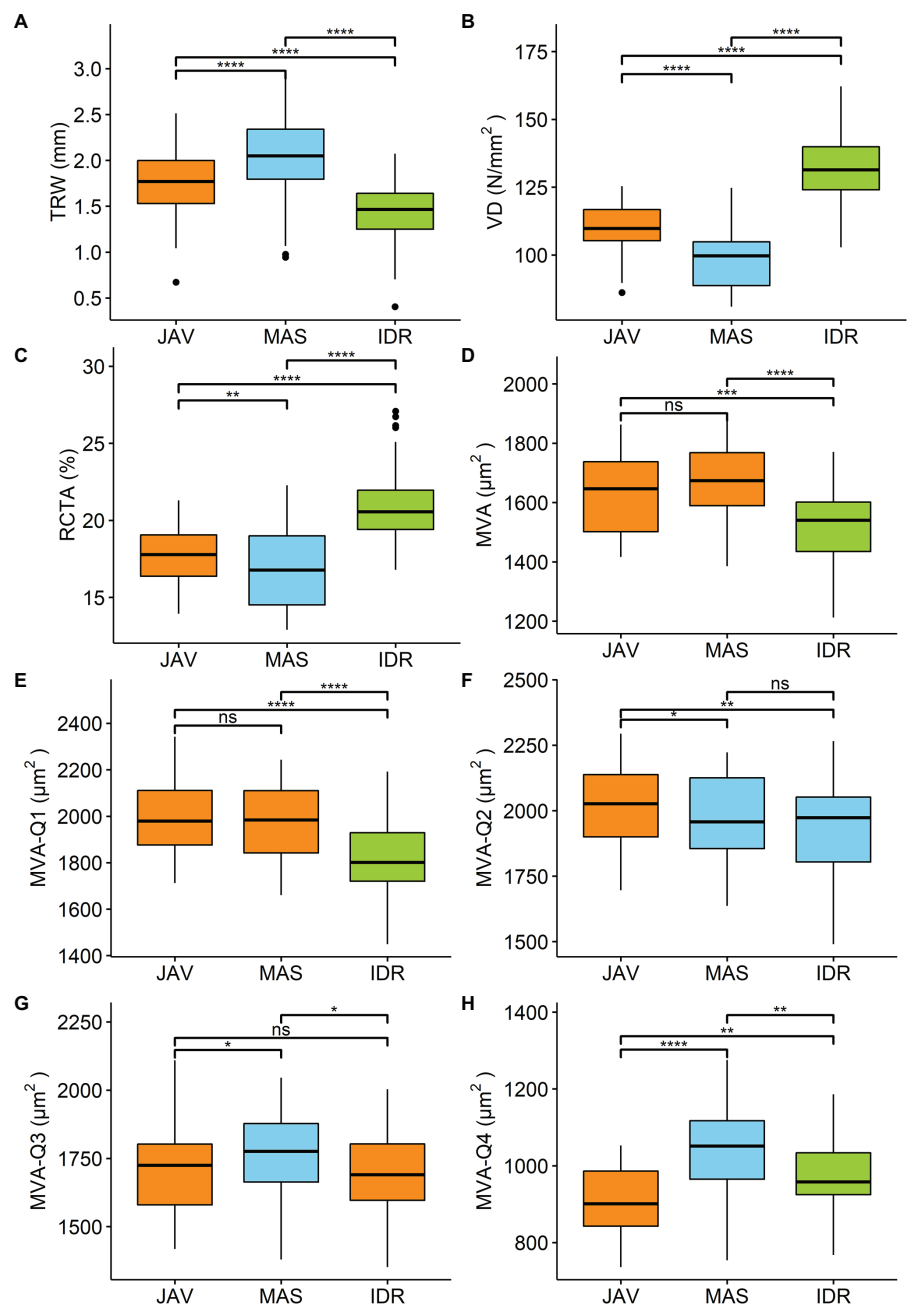
Differences among the sites Javornik (JAV), Mašun (MAS), and Idrija (IDR) in **(A)** tree ring width (TRW), **(B)** vessel density (VD), **(C)** relative conductive area (RCTA), **(D)** mean vessel area (MVA), and **(E-H)** MVA in first to fourth quarter of tree ring (MVA-Q1–MVA-Q4) analyzed by rm-ANOVA or the Friedman test. Significance of differences in tree ring characteristic between sites are market by ns-not significant. ^*^p < 0.05, ^**^p < 0.01, ^***^p < 0.001, and ^****^p < 0.0001. Additional information is available in Supplementary Table S1.

The local TRW chronologies at MAS, JAV, and IDR were between 112 and 151 years long (Table 2). The widest mean TRW for the 1960–2016 period was measured at MAS (2 ± 0.5 mm), followed by JAV (1.8 ± 0.3 mm) and IDR (1.4 ± 0.3 mm); the differences were statistically significant (Figure 2A). The measured vessel features VD and RCTA were significantly different between all sites (Figures 2B–C). MVA was similar at MAS and JAV, but significantly different at site IDR (Figure 2D).

**Table 2:** Tree ring width and wood anatomy chronologies and descriptive statistics: first-order autocorrelation (AC1), Gleichläufigkeit coefficient (%GLK), mean inter-series correlation (rbar), and expressed population signal (EPS) for the selected sites; Javornik (JAV), Masun (MAS), and Idrija (IDR).

Site		Start year	End year	AC1	%GLK	rbar	EPS
JAV	TRW	1885	2016	0.63	69	0.38	0.89
MAS		1904	2016	0.80	64	0.48	0.93
IDR		1865	2016	0.77	66	0.25	0.83
JAV	MVA	1940	2016	0.59	49	0.45	0.83
MAS		1940	2016	0.56	50	0.32	0.74
IDR		1940	2016	0.63	53	0.01	0.04
JAV	VD	1940	2016	0.63	51	0.04	0.04
MAS		1940	2016	0.73	53	0.06	0.29
IDR		1940	2016	0.61	52	0.10	0.38
JAV	RCTA	1940	2016	0.64	49	0.19	0.58
MAS		1940	2016	0.62	52	0.11	0.42
IDR		1940	2016	0.68	54	0.14	0.48
JAV	MVA-Q1	1940	2016	0.51	54	0.32	0.74
JAV	MVA-Q2	1940	2016	0.58	47	0.35	0.79
JAV	MVA-Q3	1940	2016	0.48	50	0.32	0.74
JAV	MVA-Q4	1940	2016	0.37	51	0.23	0.64
MAS	MVA-Q1	1940	2016	0.50	51	0.28	0.69
MAS	MVA-Q2	1940	2016	0.52	50	0.31	0.73
MAS	MVA-Q3	1940	2016	0.43	50	0.23	0.64
MAS	MVA-Q4	1940	2016	0.37	53	0.30	0.71
IDR	MVA-Q1	1940	2016	0.55	54	0.08	0.32
IDR	MVA-Q2	1940	2016	0.54	54	0.06	0.26
IDR	MVA-Q3	1940	2016	0.49	53	0.04	0.15
IDR	MVA-Q4	1940	2016	0.46	53	0.04	0.16

**Figure 3:**
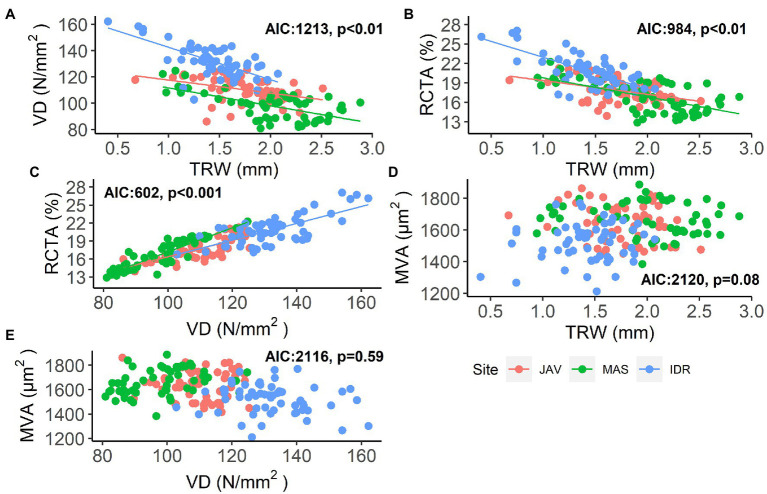
Relationships between: **(A)** vessel density (VD) and tree-ring width (TRW), **(B)** relative conductive area (RCTA) and TRW, **(C)** RCTA and VD, **(D)** mean vessel area (MVA) and TRW, and **(E)** MVA and VD in beech at Javornik (JAV), Mašun (MAS), and Idrija (IDR) with models Akaike information criterion (AIC) and significance level. Further statistical information for the presented generalized linear models is given in Supplementary Tables S2–S6.

The measured vessel traits generally exhibited significant relationships with TRW (Figure 3). At all sites, a negative relationship was observed between TRW and VD and between TRW and RCTA, indicating differences in theoretical specific conductivity and vessel densities between wider and narrower tree rings (Figures 3A,B; Supplementary Tables S2 and S3). Consequently, RCTA and VD showed a significant positive relationship at all sites (Figure 3C; Supplementary Table S4); rings with higher VD resulted in higher RCTA. As expected, neither there was no significant relationship between TRW and MVA, nor between VD and MVA (Figures 3D,E; Supplementary Tables S5 and S6).

**Figure 4:**
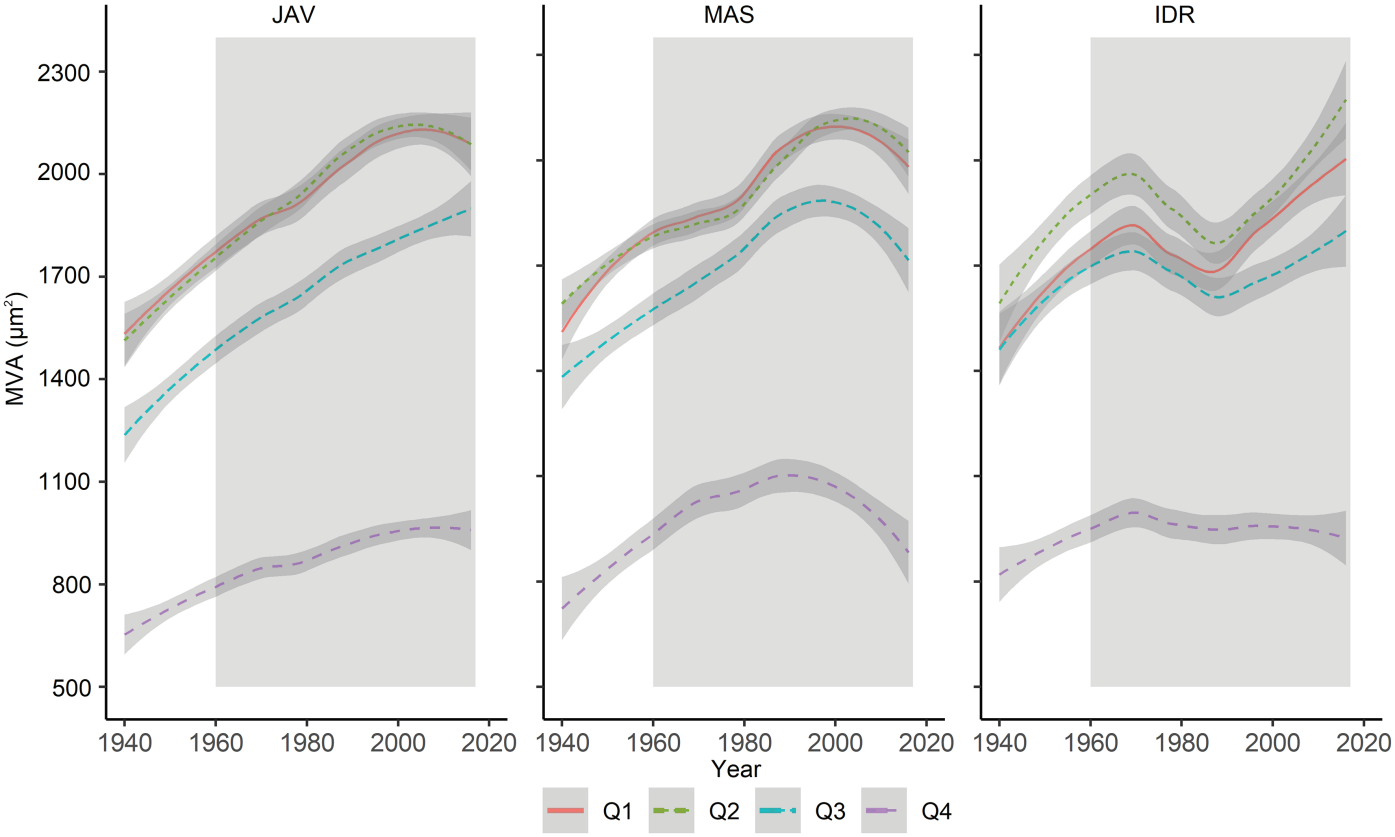
Long-term trends in MVA in evaluated tree-ring quarters (Q1–Q4) at Javornik (JAV), Mašun (MAS) and Idrija (IDR). The gray area represents the period between 1960 and 2016, which was considered for further analysis. The relationships between sectors are presented in Supplementary Figure S1.

Further comparison of MVA values in the tree-ring quarters showed a consistent decrease in vessel area from the first to the last quarter (Figure 4). In general, MVA was similar in the first and second quarters, but was smaller in the third and fourth quarters (Figures 2E–H, 4). The differences in MVA between the first and second quarters were largest at IDR, while at MAS and JAV, the values were similar (Figure 4). MVA in the third quarter was smaller than in the first two quarters at all sites. A decrease in MVA from the second to the third quarter was between 10% at MAS and 15% at JAV. An abrupt decrease in MVA was noted for the fourth quarter, where MVA at all selected sites decreased by more than 40% compared to the third quarter (Figures 2G,H; Supplementary Table S1). Nevertheless, inter-quarter correlation analyses revealed a relatively strong positive relationship between MVA in the individual tree-ring quarters at each site. At JAV correlation values were similar between quarters, while slightly lower correlation values were found between the first and last quarters at sites MAS and IDR (Supplementary Figure S1).

### The Effect of Climate Variables on Tree-Ring and Vessel Parameters

**Figure 5:**
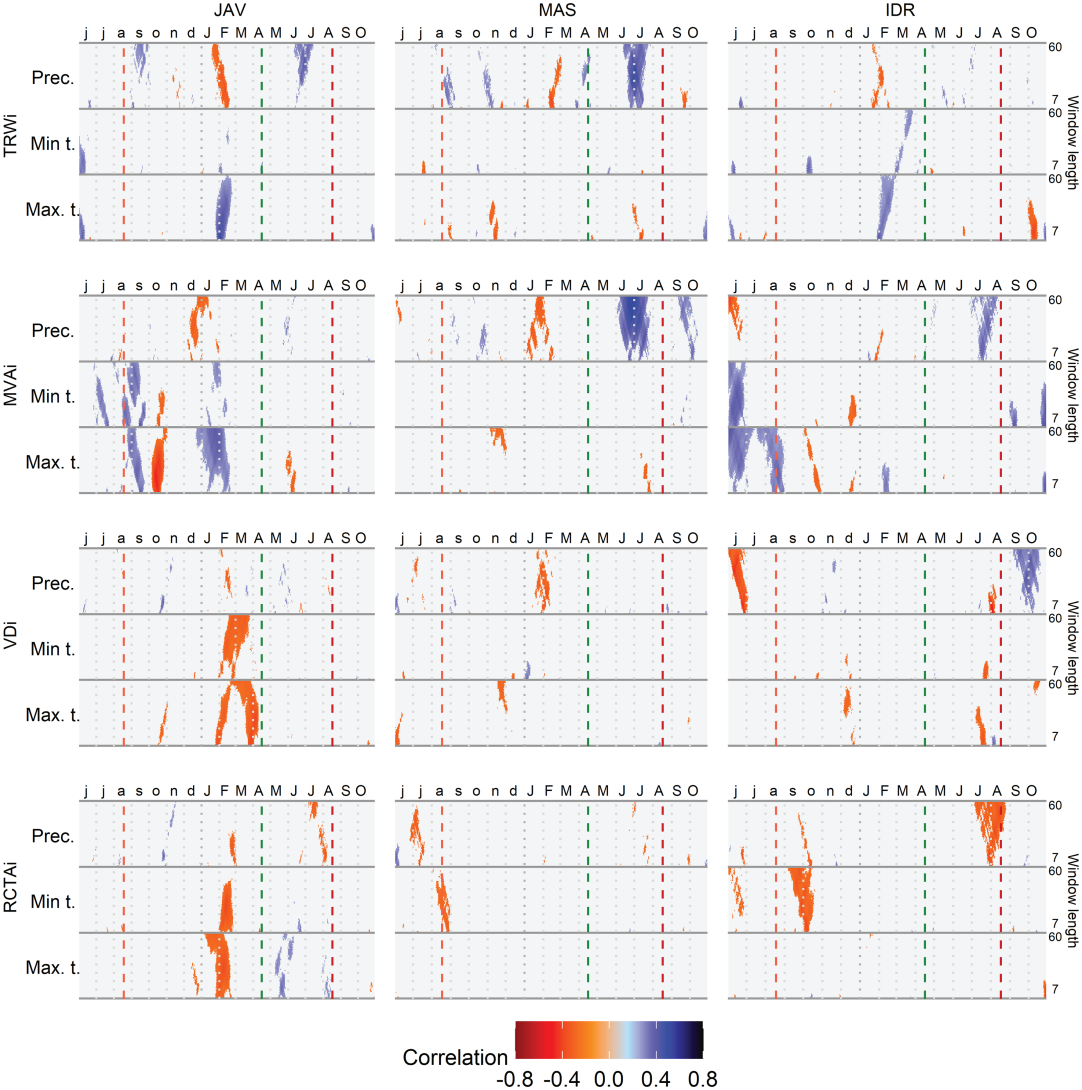
Correlations between standardized tree-ring chronologies (tree ring width - TRWi, mean vessel area - MVAi, vessel density - VDi and relative conductive area - RCTAi) and maximum and minimum daily temperature and daily precipitation sums at Javornik (JAV), Mašun (MAS) and Idrija (IDR) using a time window spanning between 7 and 60 days. Vertical dashed lines from left to right depict the approximate timing of the growing season based on previous data (Prislan et al., 2013): end of previous growing season (light red), start (green), and end (dark red) of current growing season.

Correlation analysis between long-term daily climate data and the studied tree-ring parameters showed a strong and significant association with temperature and precipitation at all sites (Figure 5). In general, the most important climatic factors related to tree-ring characteristics were amount of precipitation and maximum temperature, while minimum temperature showed lower correlations. The time window of 7–60 days showed a relatively constant climate response with the most pronounced correlations between 25 and 35 days. In the case of the MVA, high correlations were also observed for shorter time-windows (7–20 days; Figure 5).

The highest correlations were observed at JAV; the inter-annual variability of TRWi was mainly related to the conditions in late winter, when maximum temperature and precipitation showed positive (*r*_tmax_ > 0.40) and negative (*r*_prec_ < −0.30) correlations, respectively. The highest negative correlation at JAV was observed between the previous autumn maximum temperature (*r*_tmax_ > 0.40) and MVAi, while the maximum temperature at the end of winter shoved a positive correlation (*r*_tmax_ > 0.30). VDi and RCTAi were negatively correlated by both maximum and minimum temperature in late winter and early spring. Correlations between climate parameters and tree-ring features were the least pronounced at IDR, where the highest positive correlations were found between MVAi and minimum and maximum temperatures at the end of the previous growing season (*r*_tmax_ > 0.30). Furthermore, the maximum temperature at the beginning of the growing season positively correlated with TRW. While at MAS, the variability in TRWi and MVAi was mainly positively correlated with early summer precipitation.

### The Effect of Climate on Intra-Annual Vessel Area Characteristics

**Figure 6:**
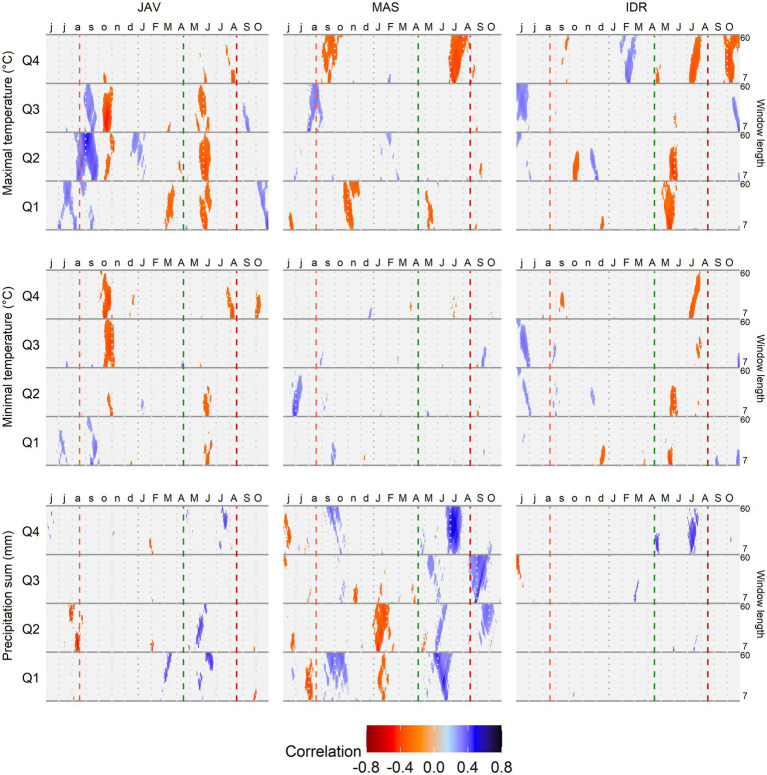
Correlation analysis between the standardized chronologies of mean vessel area (MVA) in quarters Q1–Q4 and maximum and minimum daily temperature and daily precipitation sums at Javornik (JAV), Mašun (MAS), and Idrija (IDR) using a time window spanning between 7 and 60 days. Vertical dashed lines from left to right depict the approximate timing of the growing season based on previous data (Prislan et al., 2013): end of previous growing season (light red), start (green), and end (dark red) of current growing season.

Correlation analysis revealed differences in MVAi responses to changing climatic conditions among the tree-ring quarters. In addition, differences in response to climate variations were observed among the forest sites (Figure 6). In general, precipitation and maximum temperature were the main climatic drivers significantly correlated with MVAi among quarters (Figure 6). Temporarily similar patterns of climate response among the sites were noticed for late spring temperature in the first quarter and late summer precipitation in the fourth quarter. The weakest correlation values in all quarters were observed with minimum temperature at MAS and precipitation at the site IDR, suggesting low sensitivity of MVAi to these climatic factors. Daily response functions revealed similar climate signal across 7–60 days climate window. In the case of maximum and minimum temperatures, higher signal was observed for shorter time-windows (7–20 days), while for precipitation, the highest signal was found with longer time windows, i.e., 20–35 days (Figure 6).

In the first quarter of the tree rings, the strongest correlations were observed with conditions at the end of the previous growing season and in the current year’s spring. In general, temperature negatively affected MVAi in late spring (*r*_tmax_ > 0.35), while the positive correlations were revealed at the end of the previous growing season (*r*_tmin_ > 0.30). In the same time intervals, precipitation showed the opposite correlations on MVAi than temperature. In the second quarter, precipitation before and during the growing season showed a negative (JAV: *r*_prec_ < −0.40) and positive (JAV: *r*_prec_ > 0.40; MAS: *r*_prec_ > 0.30) correlations with MVAi, respectively. Furthermore, maximum temperature showed the highest positive correlation at the end of the previous growing season at JAV (*r*_tmax_ > 0.45), although correlations were negative during the current growing season (*r*_tmax_ < −0.35). In the third quarter, correlation values were in general lower than those of the other three quarters. The exception was maximum temperature at JAV, which showed high negative correlations in the previous autumn (*r*_tmax_ < −0.50). In the fourth quarter, the highest positive correlations were observed between MVAi and summer precipitation (*r*_prec_ > 0.55), while maximum temperature during the summer revealed negative correlations on MVAi (*r*_tmax_ < −0.4). Correlation analysis also revealed a mostly reverse climatic signal between the end of the previous and the current growing seasons. At the end of the previous growing season, MVAi of all quarters of tree ring was positively and negatively correlated with temperature and precipitation, respectively. However, in the current growing season, the relationship between MVAi of all quarters and temperature and precipitation was just the opposite.

### Impact of Extreme Weather Conditions During Summer on the Studied Tree-Ring Parameters

Because late summer precipitation of the current growing season showed the highest correlation with MVA within quarters, we identified 3 years with the driest and wettest summers at the selected sites to compare intra-annual vessel area distributions between extreme wet and dry conditions. Compared to wet summers, drier summer conditions resulted in narrower TRW, higher VD, and, consequently, a higher RCTA. Differences in the studied tree-ring parameters between wet and dry summer conditions were significantly different at MAS and JAV, while beech at IDR showed smaller, insignificant differences in tree ring width parameters between extremely dry and wet years (Table 3).

**Table 3:** Differences in mean tree-ring parameters and mean summer climatic conditions among the three wettest and driest years at sites Javornik (JAV), Mašun (MAS), and Idrija (IDR).

Site	Season	Year	TRW		RCTA		VD		Extreme June – July climate							
									Prec. (mm)		Max. temp (°C)		Min. temp (°C)		SPEI	
JAV	Dry	2006; 2012; 2013	1.2	**	21.0	**	121	ns	110	***	25.1	***	11.3	**	−2.29	***
	Wet	1965; 1961; 1989	2.1		16.4		108		402		21.1		8.9		0.81	
MAS	Dry	2006; 2013; 2016	1.1	**	20.3	***	115	**	89	***	22.5	***	9.9	**	−2.44	***
	Wet	1961; 1965; 1989	2.2		14.3		85		392		18.9		7.4		0.66	
IDR	Dry	1994; 2003; 2006	1.3	ns	21.2	ns	138	ns	174	**	26.2	***	13.7	**	−1.69	***
	Wet	1995; 1980; 1990	1.5		19.8		128		543		21.0		10.8		0.89	

TRW, tree-ring width; RCTA, relative conductive area; VD, vessel density; Prec, precipitation amounts; Max. temp, maximum temperatures; Min. temp, minimum temperatures; and SPEI, standardized precipitation evapotranspiration index; JAV, MAS, and IDR. Significance of differences in tree ring characteristic and climate variables between dry and wet seasons (ANOVA) is market by ns, not significant; ^**^p < 0.01 and ^***^p < 0.001.

**Figure 7:**
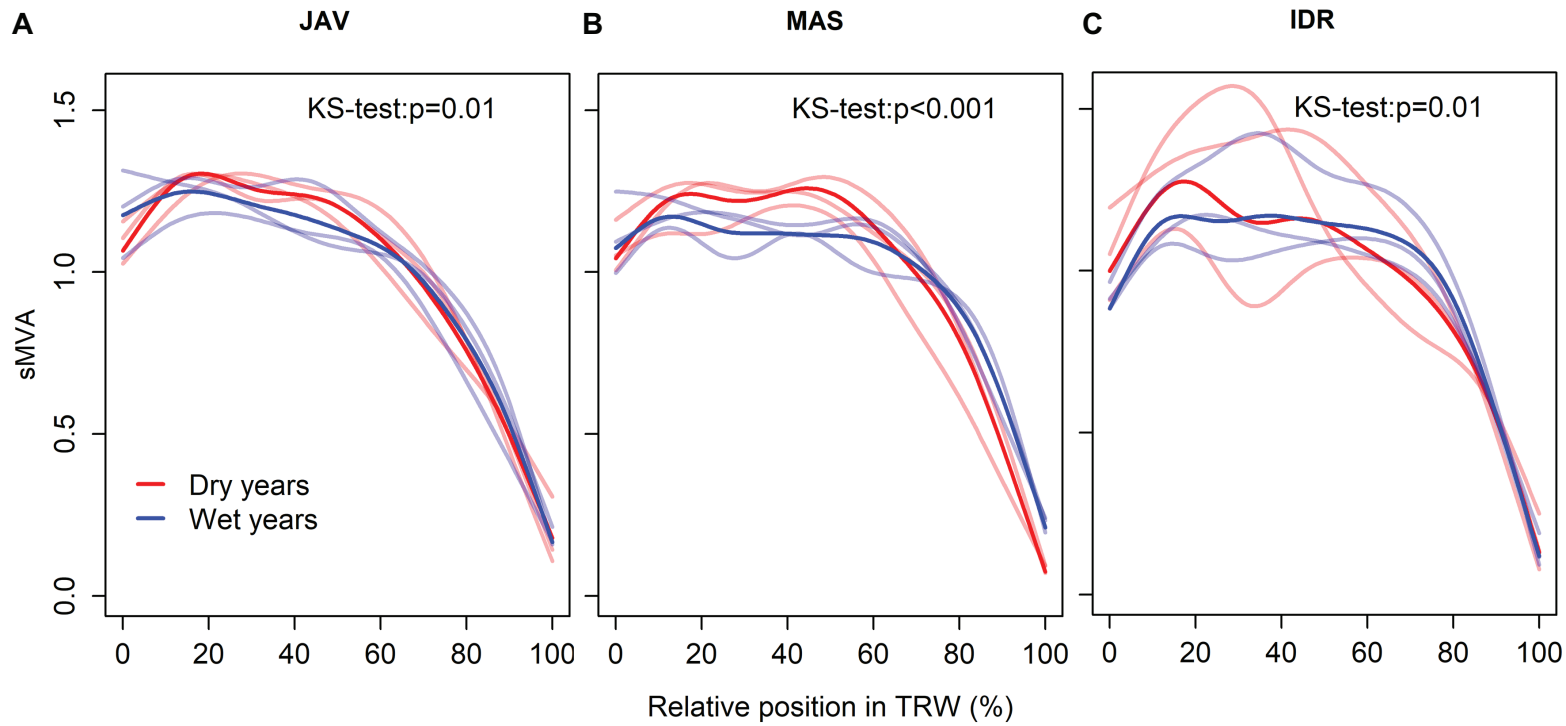
Standardized tracheograms sMVA in common beech for the wettest and the driest summer seasons at the selected sites; **(A)** Javornik (JAV), **(B)** Mašun (MAS), and **(C)** Idrija (IDR). Red and blue lines represent extreme dry and wet years, respectively. Lines with more intensive color represent the mean while transparent lines showing the variability of the selected 3 years for each extreme climate condition. The differences between general additive model (GAM) trends were tested by the Kolmogorov–Smirnov test.

In general, vessels with the largest lumen area usually appeared between 15 and 40% of the tree ring width, whereas initial vessels were slightly smaller (Figures 7A–C). Furthermore, a different declining trend in MVA was observed in the second half of the tree ring width in the case of wet and dry summers. In a wet summer, MVA slowly decreased to 60–70% of tree ring width, while an abrupt decrease in MVA was observed in the last 25% of the tree ring width. In a dry summer, MVA significantly decreased after 50% of tree ring width. At all sites, the differences in intra-annual trends between dry and wet summers were significant (Figure 7). The most pronounced differences in decreasing MVA trend were observed at MAS (Figure 7B).

## Discussion

A comparison of TRW and vessel features, and their relationships with climate factors, revealed differences between beech trees from three sites in Slovenia differing in climate conditions and leaf phenology. At all sites, a significant relationship was found between VD, RCTA, and TRW, while MVA showed no relationship with other parameters, suggesting different response to variation in temperature and precipitation. Our first hypothesis was thus confirmed. We also confirmed our second hypothesis; the response of TRW, VD, and RCTA to local climatic conditions differed between sites at the inter-annual level. Differences were also observed between sites in the MVA climatic signal at the intra-annual level. In addition, the response of MVA varied temporally within the tree ring; the first quarter showed the highest correlation with the previous year’s climatic conditions, while the last quarter showed the highest correlations with current summer conditions (confirming our third hypothesis). However, under extreme weather conditions (i.e., dry summer), trees at all sites responded similarly (i.e., narrower TRW, higher VD, and RCTA). Contrary to our expectations, trees at IDR (which is characterized as a late flushing site) showed the least pronounced response to climatic conditions compared to the other two sites, (characterized as early flushing), therefore the fourth hypothesis was rejected.

### Relationship Between Measured Tree-Ring Parameters

We developed multi-decadal chronologies of TRW and vessel features at the three forest sites representing the original stands of three Slovenian beech provenances. The comparison showed significant differences among sites in terms of TRW, VD, and RCTA. Further correlation analysis revealed that, in wider tree rings, VD and RCTA were generally smaller, which is in agreement with previous studies (Eilmann et al., 2014; Oladi et al., 2014; Diaconu et al., 2016). Since vessels of multiple years are involved in water transport in diffuse-porous beech, the influence of a single tree ring on the total water-transport capacity is only partial (Gasson, 1985). Nevertheless, higher RTCA in narrower rings suggests priority being given to water transport rather than mechanical functions, since the need for additional strength becomes less important in adult trees (Rao et al., 1997).

In contrast to VD and RCTA, MVA did not show significant relationships with TRW. Similar results for beech were reported by Sass and Eckstein (1995) and Diaconu et al. (2016), who concluded that MVA is affected by different environmental signals than TRW and can therefore be used as an additional ecological indicator. Oladi et al. (2014) suggested that VD and TRW are highly dependent on environmental factors, while MVA and RCTA are more endogenously controlled and therefore show less year-to-year variation (Carrer et al., 2015). Here, we observed that MVA was similar at JAV and MAS, while it was significantly smaller at IDR, which could be linked to differences in leaf phenology. In several genetic studies (Brus, 2010; Robson et al., 2018), the beech population at IDR was considered to be late flushing, which could be an adaptation to surviving long winters during the last glacial period. The importance of the genetic predisposition of beech has been studied in recent decades with common garden experiments (Eilmann et al., 2014; Hajek et al., 2016). Eilmann et al. (2014) reported that radial increments in beech are mostly controlled by environment, while the water transport system may reflect provenance-specific adaptation and thus genetic predisposition.

### Climate Influence on Tree-Ring Width

Trees at JAV showed the strongest response to variation in climatic conditions and trees at IDR the weakest. IDR was characterized by the highest amount of annual precipitation and had the highest average annual temperature among the sites (Table 1). Considering the findings from provenance trials (e.g., Robson et al., 2011) that IDR is a late flushing provenance, it could be assumed that radial growth at this site occurs at higher temperatures and that the trees are therefore less sensitive to fluctuations in climatic conditions (Prislan et al., 2013; Martinez del Castillo et al., 2018). Similar weak climate-growth relationships have been found for several other beech forest sites in the eastern Alps at elevations between 800 and 1,100 m a.s.l. (Dittmar et al., 2003; Di Filippo et al., 2007). This implies that precipitation is not limiting at these sites, and thus temperature mainly controls growth. Similar findings were recently shown for Norway spruce, where only temperature was found to be a limiting climatic factor at sites with more than 1,600 mm annual precipitation (Jevšenak et al., 2021).

Based on 10-year xylogenetic observations of beech from two sites in Slovenia (Prislan et al., 2019), we estimated that the growing season at the forest sites included in this study starts at the beginning of May and ends by mid-August. According to these estimates, TRW at the selected sites is mostly controlled by temperature before the growing season and current summer precipitation (Figure 5; Piovesan et al., 2005; Skomarkova et al., 2006). By comparing sites in southern and western Europe, Hacket-Pain et al. (2016) identified previous and current summer temperatures and summer precipitation as the main climatic signals affecting the growth of beech. While for northern and north-eastern part of beech distribution range, growth was limited by combination of spring frost and drought stress (Muffler et al., 2020). Finally, growth reduction in beech may also be associated with late spring frost, summer drought, or mast years, which are also strongly affected by temperature (Kolář et al., 2017; Hacket-Pain et al., 2018; Gazol et al., 2019; D’Andrea et al., 2020). Due to climate warming, a higher masting frequency of beech has been observed in recent decades in Central Europe (Bogdziewicz et al., 2021).

### Climate Influence on Vessel Features

Variation in vessel diameter is one of the most important parameters for evaluating tree-water relations. Vessel diameter determines numerous physiological xylem traits, such as hydraulic conductivity, vulnerability to freezing-induced embolism, vulnerability to drought-induced cavitation, and probably also to pathogen spread. By adjusting vessel diameter, number and distribution within annual rings, trees regulate water transport efficiency and xylem safety (Oladi et al., 2014; Gleason et al., 2016). Hajek et al. (2016) found that beech copes with drought stress by adjusting vessel number rather than vessel area. Most recent studies, however, have shown that certain tree-level properties, such as tree size and crown size, explain vessel diameter variation much more than general climatic conditions (Rosell et al., 2017). Due to basipetal vessel widening, vessel area also depends on its position in a tree (Anfodillo et al., 2012), which needs to be considered when conduit characteristics among different trees and sites are compared (Carrer et al., 2015). In our study, beech trees of comparable heights were sampled to rule out this effect on conduit properties.

The final area of vessels is influenced by endogenous (hormones and cell turgor) and environmental factors (temperature, water availability, and soil nutrients) to optimize xylem water transport from roots to leaves in terms of its safety and efficiency (Tyree and Sperry, 1989; Arend and Fromm, 2007; Hölttä et al., 2010; Hacke et al., 2017). In beech, leaf development and cambial stem reactivation in spring occur almost simultaneously (Prislan et al., 2013). Since leaf development is also environmentally controlled, this may affect the rate of photosynthetic activity and auxin production, as well as the rate of their transport within a tree. Coordination between leaf and stem vascular development therefore exists. The different timing of budburst among selected sites thus suggests that environmental conditions at the onset of radial growth differ. It can be assumed that in the case of the late flushing beech at IDR, these processes occur in the period of higher temperature compared to the early flushing beech at JAV and MAS.

There are contradicting findings in the literature regarding the dendroclimatological potential of beech vessel features (Pourtahmasi et al., 2011). Vessel features are species-specific characteristics, although external factors affect their final area to some degree. The plastic response of beech tree-ring structure in response to site conditions has been reported previously (Eilmann et al., 2014; Stojnić et al., 2015). Under favorable growing conditions, precipitation and temperature do not affect vessel characteristics (Prislan et al., 2018), while in an extremely dry year, a significant link between soil moisture and vessel area has been found (Giagli et al., 2016). Of all the measured vessel features, only MVA showed consistently high correlations with different climatic parameters at all sites, but at different time periods. While MVA at IDR and JAV showed high positive correlations with the temperature in the previous growing season, it was mainly correlated by summer precipitation at MAS. Although the annual amount of precipitation at MAS is relatively high (more than 1,600 mm), it is located in the sub-Mediterranean Karst region, on soils with low water retention capacity (Bakalowicz, 2015). Thus, trees can experience water deficit during growth, even with a relatively high amount of precipitation. The positive correlation between temperatures in summer and autumn of the previous year on vessel area at JAV and IDR could be explained by the relationship between cessation of wood formation in the previous autumn and the onset of leaf phenology in the current vegetation period, recently observed by Marchand et al. (2020). They found earlier bud-burst in the case of earlier cessation of wood formation in the previous year. Earlier bud burst and the development of leaves may be related to changes in hormone concentrations (i.e., auxin) affecting the differentiation of vessels (Aloni, 2007). The positive effect of previous autumn conditions may thus positively contribute to the amount of storage reserves, which are used for foliage development in the next spring and for maintaining winter respiration (Barbaroux et al., 2003). Furthermore, assuming sufficient water availability, we would expect more photo-assimilates in a warm summer and, consequently, greater carbohydrate reserves (and growth; Klesse et al., 2020). Greater carbohydrate reserves could affect the turgor required and thus cell size (Cabon et al., 2020). Compared to other species (e.g., pedunculate oak), the first quarter of tree ring width in beech is less dependent on stored carbohydrate reserves because its growth starts at the same time as leaf development, which quickly start to supply developing tissues with assimilates (Barbaroux and Bréda, 2002).

Although beech is a diffuse-porous species, vessel area in initial and terminal parts of the tree ring differs, being smaller in the latter part (Sass and Eckstein, 1995; Prislan et al., 2018). We found that initial vessels were smaller than the subsequent ones (Figure 7), which may reflect different environmental conditions at the time of their formation, especially in terms of moisture supply. On an intra-annual scale, no atypical distribution of vessel area (e.g., intra-annual density fluctuations – IADFs) was observed at any of the selected forest sites (Supplementary Figure S2). In the first, second, and third quarters, MVA was similar among the sites (Figure 2), while in the last quarter, MVA significantly differed among all sites, suggesting a strong link with local conditions (Pourtahmasi et al., 2011). By dividing tree rings into quarters, relationships between MVA and climate conditions were evaluated at the intra-annual level (Figure 6). To the best of our knowledge, apart from Sass and Eckstein (1995), no such analyses have been performed for beech. We found that different climatic variables generally controlled the distribution of vessel area within tree rings at the selected sites. In the first quarter, MVA negatively and positively correlated with maximum temperature and precipitation before the growing season and in spring, respectively. While, vessel area in the second quarter was negatively correlated with early summer temperatures. In addition, MVA in the last quarter was positively and negatively correlated with mid-summer precipitation and maximum temperature, respectively. A positive correlation between late summer precipitations on vessels formed at the end of the growing season was also reported by Sass and Eckstein (1995). The results suggest the intra-annual variability in vessels area could be used for reconstruction of climate conditions before and during the time they were formed (e.g., Castagneri et al., 2017). In order to better understand how short-term climate conditions during the growing season affect vessel formation, the findings should be verified with studies on intra-annual xylem formation (e.g., Prislan et al., 2018). Furthermore, the influence of intra-seasonal climate variability on other vessel features (e.g., vessel grouping or potential hydraulic conductivity) may also be evaluated (Schuldt et al., 2016).

### Beech Tree Ring Features Under Climate Change (Extreme Weather Conditions)

In the past six decades, the average annual temperature has increased by 0.36°C, and the amount of precipitation has decreased by 3% per decade in central Europe (de Luis et al., 2014; Vertačnik et al., 2018). Slovenia belongs to a transitional region, which is affected by both, Mediterranean and temperate climate regimes (Koffi and Koffi, 2008). Thus, based on different future climate scenarios further increases in temperature, changes in precipitation patterns and increased frequency of extreme weather events are expected which could result in changing climate and growth conditions (Dolschak et al., 2019). Several dendroclimatological studies have suggested that increasing temperature will positively affect radial growth at typical beech forest sites (Dulamsuren et al., 2016; Martinez del Castillo et al., 2018; Vospernik and Nothdurft, 2018; Prislan et al., 2019). However, these studies did not generally take into account the overall impact of extreme weather events on tree growth and possible changes in beech spatial distribution (Saltre et al., 2015). In addition, extreme weather events may have different effects on tree growth (Weigel et al., 2018). Climate-associated severe events, such as droughts, ice storms, and heatwaves, amplify the susceptibility of beech to secondary damage by various pathogens and pests (La Porta et al., 2008). Also in this study, we observed an unusual trend in the MVA chronology at IDR, indicating a decline in MVA around 1980 (Figure 4). Indeed, historical records show a large-scale ice break disturbance at this site (Šifrer, 1977), which seems to have affected the growth of some trees. Recent studies showed that late spring frost essentially influenced beech foliage and TRW in the current year (D’Andrea et al., 2020; Decuyper et al., 2020; Sanguesa-Barreda et al., 2021). However, trees fully recovered in the following growing season, which indicates high resilience of beech to this stress event (D’Andrea et al., 2020). In contrast, no effect of summer drought was found on the radial growth of beech in the current year (D’Andrea et al., 2020), but it may be evident in the following year (Decuyper et al., 2020).

In addition to TRW, extreme climatic events significantly affect leaf phenology and wood anatomy (Bräuning et al., 2016; Carrer et al., 2016; Popkova et al., 2018). It was found that the effect of drought on vessel diameter is more pronounced if water shortage occurs early in the growing season rather later in the season. This suggests an increased sensitivity of earlywood cells to drought conditions (Arend and Fromm, 2007; Cabon et al., 2020). However, we found that vessel features (area and distribution) in beech at the selected sites were affected by drought conditions between June and August. In dry years, the trend of vessel area already began to decrease in the first half of tree ring width, which could affect turgor, resulting in smaller vessels (Popkova et al., 2018; Cabon et al., 2020). This was particularly evident in the exceptionally dry year 2003, when an almost semi-ring porous distribution of vessels was observed (Supplementary Figure S3B) in some tree rings at IDR (Bosshard, 1982; Schweingruber, 2007). In wetter and average years, on the other hand, an abrupt decrease in vessel area is observed in the fourth quarter of the annual ring (Figure 7; Supplementary Figure S3A).

## Conclusion

This study has shown that the emerging field of quantitative wood anatomy complements both dendroecological studies and climate change research (Buttò et al., 2020; Piermattei et al., 2020). For example, vessel area and distribution, in addition to annual tree ring width, provide a better understanding of how changes in growing conditions affect not only radial growth, but also wood anatomy and its properties. Our results show differences in relationships between climate and tree ring features (e.g., TRW, VD, RCTA, and MVA) at the selected sites. We can conclude that beech trees with different leaf phenologies respond differently to changes of climatic condition, whereby early flushing beech sites are affected by similar climate drivers, while beech characterized as late flushing seems to be less sensitive to climatic changes. Although wood anatomical traits, such as vessel diameter and frequency, are easily accessible ecological or climatological proxies for analyzing adaptation processes to environmental changes (Campelo et al., 2010; Crous et al., 2012), more detailed knowledge is needed on how much of the variability in these traits is environmentally driven (Leal et al., 2003; Fisher et al., 2007; Tixier et al., 2013). In order to better understand the response of beech with different leaf phenology and growth strategy, additional sites from elevational transect and variable soil and stand properties, as well as ecophysiological measurements should be included in future analyses.

## Data Availability Statement

The raw data supporting the conclusions of this article will be made available by the authors, without undue reservation.

## Author Contributions

PP, JJ, JG, and DA planned and designed the research. PP and GB performed the sampling. DA and PP contributed to the sample preparation and capturing of high resolution images with light microscope and wrote the manuscript with contributions from all co-authors. DA and GA contributed to image analyzing in Roxas and Image-Pro Plus. DA, JJ, and PP analyzed and interpreted the data. All authors contributed to the article and approved the submitted version.

## Conflict of Interest

The authors declare that the research was conducted in the absence of any commercial or financial relationships that could be construed as a potential conflict of interest.

The reviewer IG-G is currently organizing a Research Topic with one of the author GA. The review process met the standards of a fair and objective review.

## Publisher’s Note

All claims expressed in this article are solely those of the authors and do not necessarily represent those of their affiliated organizations, or those of the publisher, the editors and the reviewers. Any product that may be evaluated in this article, or claim that may be made by its manufacturer, is not guaranteed or endorsed by the publisher.
