# Supplementary material for: Different Wood Anatomical and Growth Responses in European Beech (Fagus sylvatica L.) at Three Forest Sites in Slovenia
Source: Front Plant Sci. 2021 Jul 26;12:669229. doi: 10.3389/fpls.2021.669229 (PMC8349990; doi:10.3389/fpls.2021.669229)
Supplement: Supplementary file 1 [file Data_Sheet_1.docx]

Supplementary Material

# Supplementary Figures and Tables

## Supplementary Figures


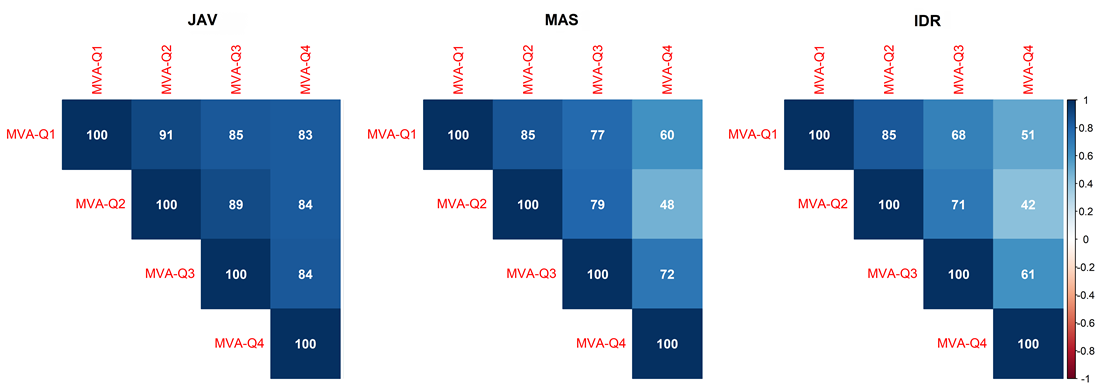


**Supplementary Figure 1.** Correlation values between mean vessel area (MVA) in the individual tree ring quarters (MVA-Q1 – Q4) at sites Javornik (JAV), Mašun (MAS) and Idrija (IDR).


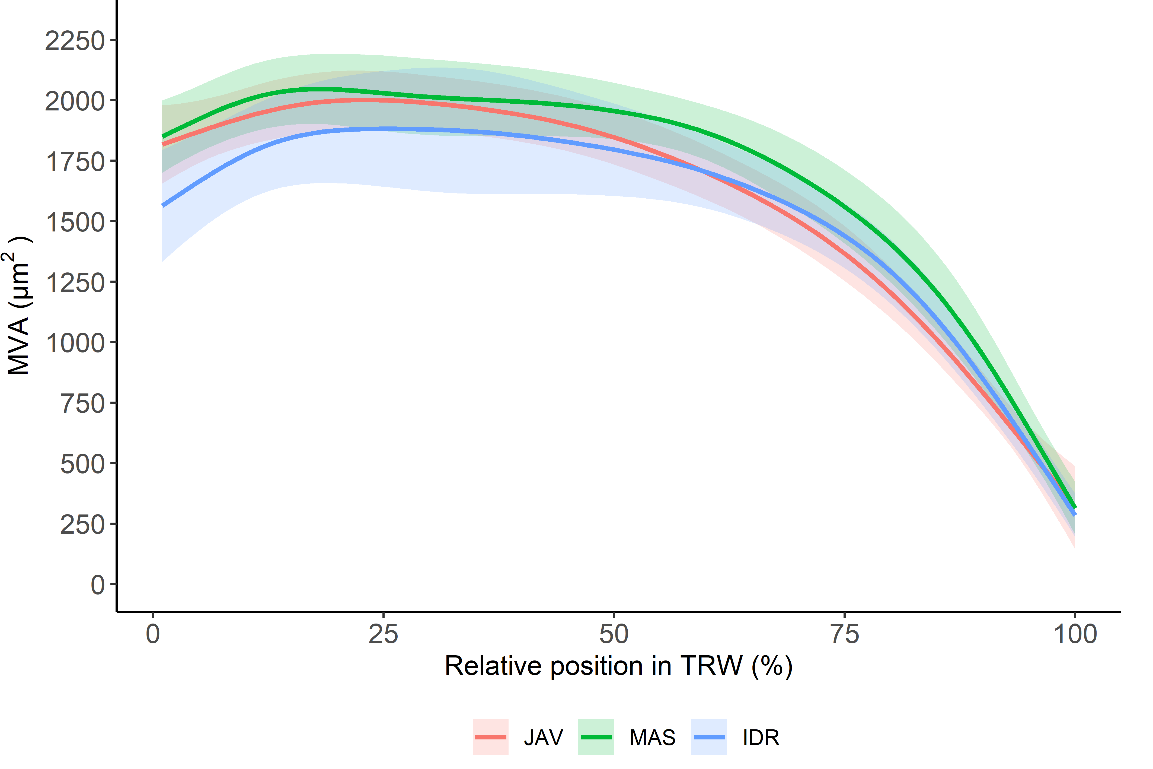


**Supplementary Figure 2.** Average beech tracheograms (GAM - General additive models) at Javornik (JAV), Mašun (MAS) and Idrija (IDR) for the period between 1960 and 2017.


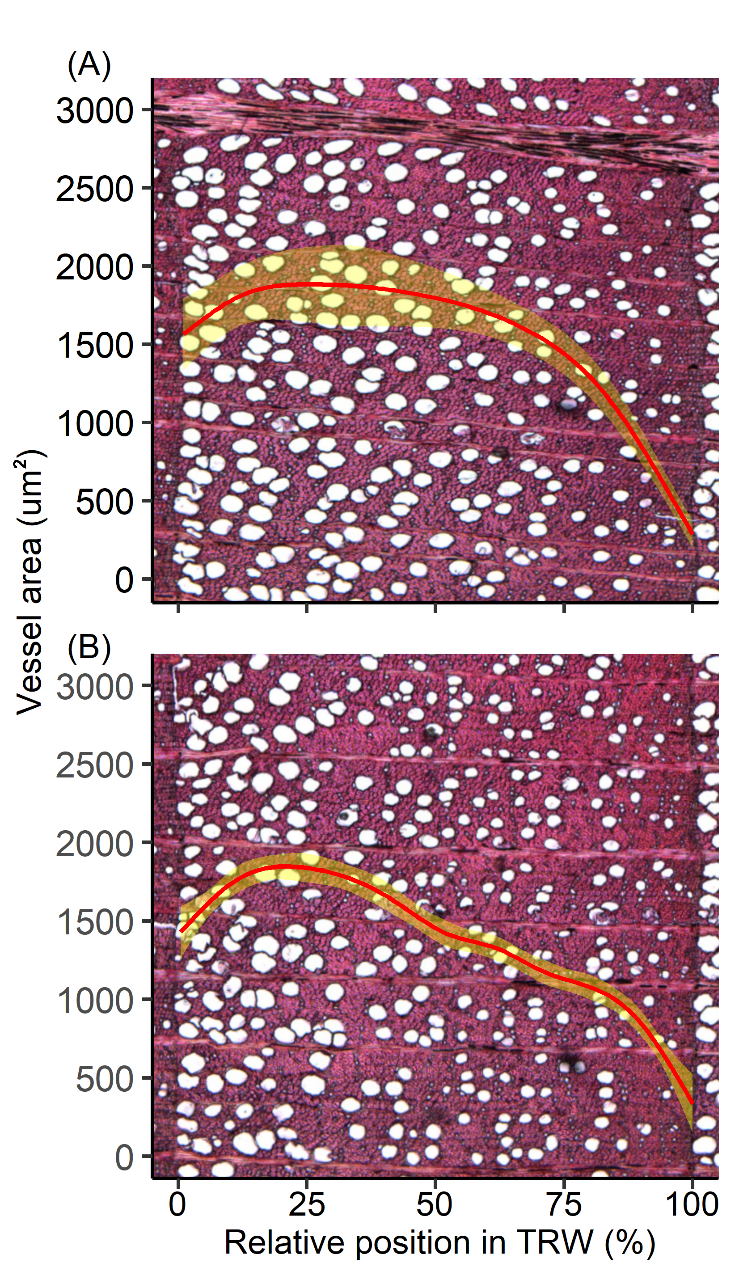


**Supplementary Figure 3.** Intra annual vessel distribution at Idrija (IDR) under average weather conditions (A) and extreme weather conditions; i.e. summer drought in 2003 (B) where almost a semi ring-porous vessel distribution was found as a response to extreme dry summer conditions.


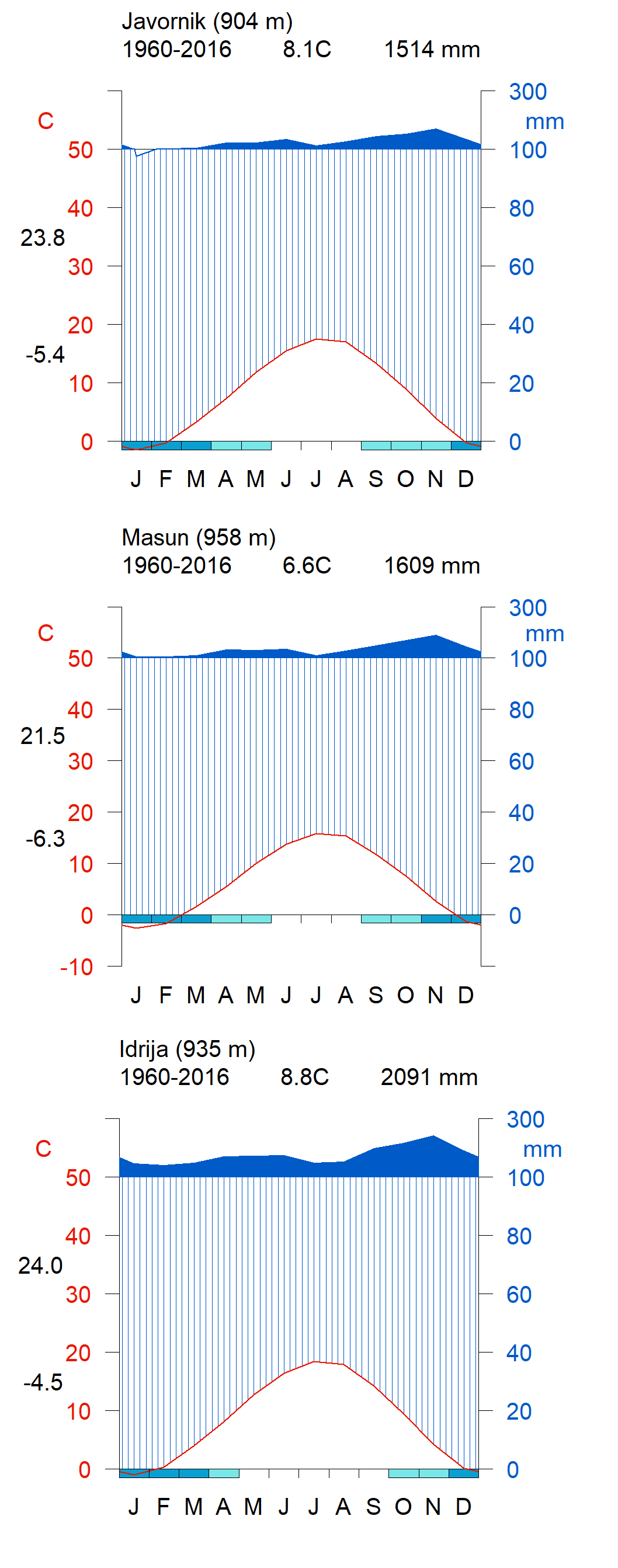


**Supplementary Figure 4.** Walter and Leith climate diagrams for selected sites Javornik, Mašun and Idrija. Figures representing a period between 1960 and 2016.

1. **Supplementary Tables**

**Supplementary Table 1**: Differences between the sites in tree ring width (TRW) and vessel chronologies (i.e., vessel density (VD), relative conductive area (RCTA), mean vessel area (MVA)) analyzed by rm-ANOVA or Friedman test. To assess the intra-annual variability, MVA was analyzed within tree ring quarters (MVA-Q1 – MVA-Q4).

| Tree ring characteristic | Test | Site ID | Mean | St. dev | Site | Pair test group |
| --- | --- | --- | --- | --- | --- | --- |
|  |  | IDR | 1.4 | 0.34 |  | c |
| TRW (mm) | Fried. t.; χ^2^= 74.8 | MAS | 2 | 0.49 | p<0.001 | a |
|  |  | JAV | 1.8 | 0.34 |  | b |
|  |  | IDR | 132 | 12.3 |  | a |
| VD (N/mm^2^) | ANOVA; F = 171,05 | MAS | 98.1 | 10.6 | p<0.001 | c |
|  |  | JAV | 110 | 8.8 |  | b |
|  |  | IDR | 20.8 | 2.4 |  | a |
| RCTA (%) | Fried. t.; χ^2^= 64.9 | MAS | 16.8 | 2.4 | p<0.001 | c |
|  |  | JAV | 17.8 | 1.8 |  | b |
|  |  | IDR | 1522 | 123.0 |  | b |
| MVA (µm^2^) | ANOVA; F = 23.0 | MAS | 1668 | 106.4 | p<0.001 | a |
|  |  | JAV | 1635 | 122.1 |  | a |
|  |  | IDR | 1819 | 157 |  | b |
| MVA-Q1 (µm^2^) | ANOVA; F = 36.7 | MAS | 1972 | 147 | p<0.001 | a |
|  |  | JAV | 2004 | 159 |  | a |
|  |  | IDR | 1922 | 173.5 |  | a |
| MVA-Q2 (µm^2^) | ANOVA; F = 6.8 | MAS | 1973 | 162.1 | p<0.01 | a |
|  |  | JAV | 2015 | 156.4 |  | b |
|  |  | IDR | 1702 | 132.1 |  | b |
| MVA-Q3 (µm^2^) | ANOVA; F = 4.8 | MAS | 1773 | 137.0 | p<0.05 | a |
|  |  | JAV | 1717 | 153.9 |  | b |
|  |  | IDR | 967 | 94.3 |  | b |
| MVA-Q4 (µm^2^) | Fried. t.; χ^2^= 34.84 | MAS | 1035 | 116.9 | p<0.001 | a |
|  |  | JAV | 906 | 83.4 |  | c |

**Supplementary Table 2**: Statistical information about regression models in Figure 3a

| glm(formula = VD ~ TRW * site) | | | | AIC:1213.4 | | |
| --- | --- | --- | --- | --- | --- | --- |
|  | Estimate | Std Error | T value | | Pr(>\|t\|) | siq |
| Intercept | 127.578 | 5.655 | 22.562 | | <0.0001 | *** |
| TRW | -9.939 | 3.163 | -3.142 | | 0.0019 | ** |
| Site-MAS | -2.548 | 7.293 | -0.349 | | 0.73 |  |
| Site-IDR | 40.720 | 7.400 | 5.502 | | <0.0001 | *** |
| TRW:Site-MAS | -3.487 | 3.871 | -0.901 | | 0.36 |  |
| TRW:Site-IDR | -15.297 | 4.534 | -3.374 | | 0.0009 | *** |

**Supplementary Table 3**: Statistical information about regression models in Figure 3b.

| glm(formula = RCTA ~ TRW * site) | | | | AIC:983.8 | | |
| --- | --- | --- | --- | --- | --- | --- |
|  | Estimate | Std Error | T value | | Pr(>\|t\|) | siq |
| Intercept | 21.49 | 1.2018 | 17,886 | | <0.0001 | *** |
| TRW | -2.1220 | 0.6723 | -3.156 | | 0.0019 | ** |
| Site-MAS | 1.2233 | 1.5500 | 0.789 | | 0.43 |  |
| Site-IDR | 6.2697 | 1.572 | 3.986 | | <0.0001 | *** |
| TRW:Site-MAS | -0.8128 | 0.8228 | -0.988 | | 0.32 |  |
| TRW:Site-IDR | -2.7384 | 0.9635 | -2.842 | | 0.005 | ** |

**Supplementary Table 4**: Statistical information about regression models in Figure 3c

| glm(formula = RCTA ~ VD * site) | | | | AIC:602.09 | | |
| --- | --- | --- | --- | --- | --- | --- |
|  | Estimate | Std Error | T value | | Pr(>\|t\|) | siq |
| Intercept | 2.7725 | 2.31 | 1.199 | | 0.2323 |  |
| VD | 0.136 | 0.0209 | 6.506 | | <0.0001 | *** |
| Site-MAS | -5.5976 | 2.877 | -1945 | | 0.05 |  |
| Site-IDR | -0.081 | 3.108 | -0.262 | | 0.79 |  |
| TRW:Site-MAS | 0.0641 | 0.0272 | 2.361 | | 0.019 | * |
| TRW:Site-IDR | 0.0064 | 0.0261 | 0.0246 | | 0.806 |  |

**Supplementary Table 5**: Statistical information about regression models in Figure 3d

| glm(formula = MVA ~ TRW * site) | | | | AIC:2120.3 | | |
| --- | --- | --- | --- | --- | --- | --- |
|  | Estimate | Std Error | T value | | Pr(>\|t\|) | siq |
| Intercept | 1775.21 | 80.16 | 22.145 | | <0.0001 | *** |
| TRW | -79.76 | 44.84 | -1.779 | | 0.08 |  |
| Site-MAS | -41.25 | 103.39 | -0.399 | | 0.69 |  |
| Site-IDR | -326.14 | 104.91 | -3.109 | | <0.002 | ** |
| TRW:Site-MAS | 46.96 | 54.88 | 0.856 | | 0.393 |  |
| TRW:Site-IDR | 130.86 | 64.27 | 2.036 | | 0.04 | * |

**Supplementary Table 6**: Statistical information about regression models in Figure 3e

| glm(formula = MVA ~ VD * site) | | | | AIC:2116.8 | | |
| --- | --- | --- | --- | --- | --- | --- |
|  | Estimate | Std Error | T value | | Pr(>\|t\|) | siq |
| Intercept | 1740.45 | 193.93 | 8.974 | | 8.974 | *** |
| VD | -0.9548 | 1.7553 | -0.544 | | 0.5872 |  |
| Site-MAS | -433.85 | 241.26 | -1.798 | | 0.074 |  |
| Site-IDR | 40.06 | 260.60 | 0.154 | | 0.88 |  |
| TRW:Site-MAS | 4.64 | 2.279 | 2.036 | | 0.04 | * |
| TRW:Site-IDR | -0.99 | 2.1914 | -0.456 | | 0.6489 |  |
